# *Cryptosporidium parvum* protease INS6 plays an important role in parasite proliferation and pathogenicity

**DOI:** 10.1371/journal.pntd.0013532

**Published:** 2025-09-12

**Authors:** Wei He, Zuwei Yang, Jing Wang, Fuxian Yang, Na Li, Rui Xu, Songrong Zeng, Lihua Xiao, Yaoyu Feng, Yaqiong Guo

**Affiliations:** 1 State Key Laboratory for Animal Disease Control and Prevention, Center for Emerging and Zoonotic Diseases, College of Veterinary Medicine, South China Agricultural University, Guangzhou, China; 2 School of Biology and Agriculture, Shaoguan University, Shaoguan, China; Tehran University of Medical Sciences, IRAN, ISLAMIC REPUBLIC OF

## Abstract

**Background:**

*Cryptosporidium parvum* is a common protozoan pathogen responsible for moderate to severe diarrhea in humans and animals. Parasite invasion and egress cause damage to intestinal epithelial cells, which is mediated by a variety of secretory proteins from several unique organelles, such as micronemes. Previous spatial proteomic analysis has identified insulinase-like protease 6 (INS6) as a putative microneme protein in *C. parvum*. However, the functional contribution of INS6 to *Cryptosporidium* pathogenicity remains poorly characterized. In this study, we used genetic manipulation techniques to investigate the expression and biological functions of INS6 in *C. parvum*.

**Methodology/principal findings:**

The *INS6* gene was tagged and deleted in *C. parvum* using CRISPR/Cas9 technology. The expression of *INS6* was determined by immunofluorescence analysis, ultrastructure-expansion microscopy, and immunoelectron microscopy. Endogenous labelling showed low levels of INS6 expression, which is found in *C. parvum* micronemes and is absent during the male gamont stage. The effect of *INS6* deletion on parasite growth and pathogenicity was assessed *in vitro* using HCT-8 cultures and *in vivo* by infection of interferon-γ knockout mice. Deletion of the *INS6* gene impaired *C. parvum* proliferation *in vitro* and significantly reduced the parasite burden in infected mice. In addition, mice infected with the *Δins6* strain showed a significant reduction in the intestinal villus-to-crypt ratio, attenuated body weight loss and increased survival rates, compared to those infected with the INS6–3HA strain.

**Conclusions/significance:**

These results indicate that INS6 protein is involved in *C. parvum* proliferation and plays a critical role in modulating the pathogenicity of this zoonotic parasite. Deletion of this gene affects the invasion efficiency and pathogenicity of the parasite.

## Author summary

*Cryptosporidium parvum* is a major cause of moderate to severe diarrhea in humans and animals. Invasion and egress of *C. parvum* are accomplished by a variety of secretory proteins from several unique organelles, such as micronemes. Previous spatial proteomics analysis has identified insulinase-like protease 6 (INS6) as a putative microneme protein in *C. parvum*. In this study, we used genetic manipulation techniques to investigate the expression patterns and functional roles of INS6 in *C. parvum* subtype IIdA20G1-HLJ, a pandemic strain that represents the predominant pathogenic variant in China. Current experimental results suggest that the subcellular localization of the INS6 protein differs from that of conventional microneme proteins. However, due to its low transcriptional level, the precise localization of INS6 cannot be fully determined. Deletion of the *INS6* gene affected *C. parvum* proliferation *in vitro* and significantly reduced the parasite burden in infected mice. This resulted in significant decreases in intestinal villus length/height ratio and body weight and prolonged survival time in infected mice. Compared with wild-type strain-infected mice, those infected with the *Δins6* strain demonstrated significant increases in villus-to-crypt ratios, body weight, and survival time. These findings collectively demonstrate that INS6 plays an important role in modulating the pathogenicity of this zoonotic parasite.

## Introduction

*Cryptosporidium* spp. are intracellular apicomplexan parasites and the leading cause of moderate-to-severe diarrhea in humans and animals [1]. Children, neonatal animals and immunocompromised individuals are particularly susceptible to *Cryptosporidium* [2]. According to the 2016 Global Burden of Disease Study, cryptosporidiosis is responsible for ~57,000 deaths and 4.2 million disability-adjusted life-years across all age groups [3]. The risk of death is highest in young children aged 12–23 months [4]. Despite the global importance of this pathogen, nitazoxanide, the only FDA-approved drug, is ineffective in malnourished and immunocompromised patients [5]. A major reason for the lack of effective drugs is the limited understanding of the mechanisms of invasion and development of *Cryptosporidium* spp. [6].

Proteases in secretory organelles are known to be involved in processing invasion-associated proteins and modifying host cell activities, and thus play an important role in host cell invasion by apicomplexan parasites [7]. Recently, a spatial proteomic analysis of *C. parvum* has identified many secretory effectors of the parasite and their biogenesis in invasion-related organelles [8]. Among them, microneme proteins are first secreted during the invasion of enterocytes. In total, 21 microneme proteins were identified, including several known to be involved in host cell invasion, such as CP23, GP60 (GP40/15), GP900 and MIC1 (TSP8) [8]. The candidate microneme proteins MIC2 (TSP4) and insulinase-like protease 6 (INS6) were recently identified through the analysis of spatial transcriptomics and single-cell RNA sequencing data [8,9]. However, only MIC2 (TSP4) was localized to the micronemes by ultrastructure-expansion microscopy (U-ExM) [8].

It was previously shown that *C. parvum* INS6 is a homolog of *Toxoplasma gondii* toxolysin 4 (TLN4), an M16A metalloprotease that is expressed in micronemes of *T. gondii* and plays a role in host cell invasion [10,11]. Polyclonal antibodies raised against recombinant INS6 reacted with the apical end of *C. parvum* sporozoites, consistent with the predicted microneme localization. However, we were unable to demonstrate a neutralizing effect of the antibodies on parasite growth [12].

In this study, we used CRISPR/Cas9 and U-ExM to investigate the expression and function of INS6 in *C. parvum*. The results confirm its expression and further suggest that INS6 is post-translationally processed and is involved in parasite proliferation *in vitro* and *in vivo*. In addition, deletion of the *INS6* gene reduced infection intensity and pathogenicity and prolonged the survival of infected mice.

## Materials and methods

### Ethics statement

All animal experiments were performed in accordance with the guidelines and the approval of the Institutional Animal Care and Use Committee, South China Agricultural University (Approval No. 2011C104).

### Maintenance of mice

Interferon-γ knockout (*Ifng*^−/−^) mice of the C57BL/6-J strain were obtained from the Institute of Laboratory Animal Science, Chinese Academy of Medical Sciences and subsequently bred at the Laboratory Animal Center of South China Agricultural University. For experimental purposes, 3–5-week-old male and female mice were randomly allocated into groups at a 1:1 ratio based on body weight, either for the generation and passage of stable transgenic *C. parvum* strains or for comparative analysis of oocyst shedding of INS6–3HA and *Δins6 C. parvum* strains. The mice were maintained in micro-isolators (Tecniplast Inc., Shanghai, China) and provided with sterile feed and filtered tap water. During infection experiments, mice inoculated with transgenic strains were administered paromomycin sulfate salt (Sigma-Aldrich, St. Louis, MO, USA) in drinking water at a concentration of 16 g/L. Throughout the study, mice were regularly monitored for body weight, coat condition, posture, and activity levels. Humane endpoints were strictly observed, and mice exhibiting a body weight loss exceeding 20% or showing signs of nonambulatory behavior were euthanized.

### Bioinformatic analysis of INS6

The amino acid sequences of *C. parvum* INS6 were retrieved from CryptoDB (https://cryptodb.org/cryptodb/app). Functional domains within the predicted amino acid sequences were identified using SMART (Simple Modular Architecture Research Tool) [13].The expression profiles of typical micronemal proteins, including the INS6, were analyzed based on a previously transcriptomic study of *C. parvum in vitro* (NCBI under BioProject No. PRJNA1011005) [14]. Briefly, RNA was extracted from free sporozoites immediately after oocyst shedding. *C. parvum*-infected host cells were collected at 3–72 hours post-infection. All samples then underwent Illumina transcriptome sequencing (Genedenovo Biotech, Guangzhou, China). RSEM (v1.3.1, https://github.com/deweylab/RSEM.git) was then used to calculate FPKM values of microneme genes, and expression was visualised using R package ‘ggplot2’ and Manhattan plots using R package ‘qqman’.

### Oocyst preparation and excystation

*Cryptosporidium parvum* IIdA20G1-HLJ isolate was obtained from a dairy farm in Heilongjiang, China [15]. The infectivity and pathogenicity of the isolate were assessed using an *Ifng*^−/−^ mouse model. The median infective dose (ID_50_) was one oocyst. Without intervention, infected mice shed 10⁷ oocysts per gram of feces for up to 60 days [16]. Treatment *Ifng*^−/−^ mice infected with transgenic strains with paromomycin resulted in mice succumbing to the infection within two to four weeks [17]. The isolate was maintained in newborn male Holstein calves sourced from a *C. parvum*-free farm as previously described [18]. Prior to infection, purified oocysts were treated with 10% Clorox (0.52% sodium hypochlorite) on ice for 10 min. The treated oocysts were then washed three times with PBS and resuspended in 1% BSA-PBS (wt/vol). To obtain free sporozoites, oocysts were excysted by incubation at 37°C for 60 min in the presence of 0.75% taurocholic acid.

### Primers

All primers were synthesized by Sangon Biotech (Sangon, Shanghai, China) and listed in S1 Table in the supplemental material.

### Endogenous gene tagging with CRISPR/Cas9

To obtain a single guide RNA (sgRNA) plasmid for tagging of the *INS6* gene, we designed the sgRNA using the eukaryotic pathogen CRISPR guide RNA/DNA design tool (http://grna.ctegd.uga.edu) to target the *C. parvum* IIdA20G1 genome, ensuring minimal off-target effects. The selected sgRNA targets a region 181 bp upstream of the stop codon in the *INS6* gene (cdg2_4270). The plasmid pACT1:Cas9-GFP, U6:sgINS6 was constructed using Gibson assembly as described [19,20]. For the tagging plasmid, a fragment encompassing the *INS6* C-terminus (994 bp) including the adjacent mutant protospacer motif and the *INS6* 3’ UTR (952 bp) was amplified from *C. parvum* genomic DNA. The 3HA-Nluc-P2A-neo reporter and pUC19 backbone were amplified from the plasmid pINS1–3HA-Nluc-P2A-neo [21]. The final tagging plasmid, pINS6–3HA-Nluc-P2A-neo, was generated by Gibson assembly of the above fragments.

### Gene depletion with CRISPR/Cas9

The Cas9 plasmid containing the sgRNA targeting *INS6* was used to generate *INS6* knockout (*Δins6*) mutants, as described above. To construct the *Δins6* mutants, a targeting plasmid, pINS6-GFP-Nluc-P2A-neo-INS6, were generated by incorporating homologous arms (933 bp upstream and 990 bp downstream of *INS6*) using Gibson assembly.

### Selection and amplification of transgenic parasites

Sporozoites released from 2 × 10^7^ oocysts were electroporated with the respective Cas9 plasmid and targeting plasmid as previously described [22]. The electroporated sporozoites were diluted with 200 µL sterile PBS and administered to *Ifng*^−/−^ mice via oral gavage. Prior to administration, the gastric acid of the mice was neutralized by oral administration of 8% sodium bicarbonate for 5 min. To select for the transgenic strain, paromomycin (16 g/L) was continuously administered in the drinking water starting 18 hours post-inoculation. Fecal pellets were collected at 3 days post-infection (dpi) and stored at 4°C for luciferase assay or purification. Oocysts were purified from the fecal pellets using discontinuous sucrose and cesium chloride gradients as previously described [23]. For passaging transgenic parasites or monitor parasite burden, 3–5-week-old *Ifng*^−/−^ mice were orally gavaged with 1000 purified transgenic oocysts per mouse. Fecal pellets were collected and stored at 4°C for further analysis.

### PCR validation of transgenic parasite strains

Genomic DNA was extracted from 100 mg of fecal material using the Fast DNA Spin Kit for Soil (MP Biomedical, Santa Ana, CA, USA). To confirm the successful insertion of the target sequence into the *INS6* gene locus, PCR was performed on the extracted fecal DNA using Phanta Max Super-Fidelity DNA Polymerase (Vazyme, Nanjing, China). The sequences of the primers used to confirm the correct 5’ and 3’ integration after homologous recombination for the *Δins6* and INS6–3HA strains are listed in S1 Table.

### Measurement of parasite burden by luciferase assay

Fecal luciferase activity was measured using the Nano-Glo Luciferase Assay (Promega, Madison, WI, USA) as described [19]. Briefly, weighed fecal pellets, ten 3-mm glass beads (ThermoFisher Scientific, Waltham, MA, USA), and 1 ml fecal lysis buffer (50 mM Tris-HCI pH 7.6, 2 mM DTT, 2 mM EDTA, 10% glycerol, and 1% Triton X-100) in a microcentrifuge tube were vortexed for 30 s in a FastPrep instrument (MP Biomedical, Santa Ana, CA, USA). After centrifugation at 15,000 × *g* for 3 min, 50 µL of supernatant and 50 µL of Nano-Glo luciferase substrate mix (diluted 1:50 in Nano-Glo luciferase substrate buffer; Promega) in each well of a 96-well plate (Corning, Corning, NY, USA). The plate was incubated at room temperature for 3 min, and luminescence was measured using a BioTek Synergy H1 Hybrid plate reader (BioTek, Winooski, VT, USA).

### Indirect immunofluorescence assay (IFA)

To assess INS6 expression in *C. parvum*, transgenic sporozoites were resuspended in PBS and dried on poly-L-lysine treated slides (Waterborne, New Orleans, LA, USA). Sporozoites were also used to infect HCT-8 cells grown on coverslips for 24 or 48 h. Slides and coverslips were fixed with 4% paraformaldehyde for 15 min and permeabilized with 0.5% Triton X-100 in PBS for 30 min. After blocking with 1% BSA at room temperature for 1 h, the slides and coverslips were incubated overnight at 4°C with mouse anti-EF1α antibody (diluted 1:1000) and rabbit anti-HA antibody (Cell Signaling Technology, Danvers, MA, USA; diluted 1:800) in 1% BSA-PBS. Following primary antibody incubation, samples were stained with Alexa Fluor 594-conjugated goat anti-rabbit IgG (diluted 1:400), Alexa Fluor 488-conjugated goat anti-mouse IgG (Cell Signaling Technology; diluted 1:400), and *Vicia Villosa Lectin* (VVL, Vector Laboratories; diluted 1:1000) for 1 h at room temperature. Nuclei were stained with Hoechst 33342 (ThermoFisher Scientific) for 15 min at room temperature. After three washes with PBS, the slides and coverslips were mounted with anti-fade mounting medium (Boster, Wuhan, China) and examined by fluorescence microscopy using a BX53 microscope (Olympus, Tokyo, Japan) or a STELLARIS 5 (Leica Microsystems, Wetzlar, Germany). Images were processed using ZEN 2 software (Zeiss).

### Western blot analysis

Purified INS6–3HA oocysts (3 × 10^6^) were treated with bleach, washed with cold PBS, and resuspended in lysis buffer (ThermoFisher Scientific) supplemented with protease inhibitors (Sigma-Aldrich). After overnight incubation at 4°C, the suspension was centrifuged at 15,000 × *g* for 3 min, mixed with protein loading buffer, and boiled for 10 min. Proteins in the lysate were separated by SDS-PAGE and transferred to a PVDF membrane. The membrane was blocked with 1% non-fat milk overnight at 4°C, then incubated with mouse anti-HA primary antibody (Cell Signaling Technology; diluted 1:1000) for 1 h, followed by HRP-conjugated anti-mouse IgG (H&L) (Sigma-Aldrich; diluted 1:10,000) as the secondary antibody. After washing three times with PBST, the membrane was treated with High-sig ECL Western Blotting Substrate and developed on a Tanon 5200 (Tanon, Shanghai, China). The membrane was subsequently stripped and reprobed with rabbit anti-cgd8_10 (encoding an uncharacterized protein of *C. parvum*; diluted 1:2,000) [24] and HRP-conjugated goat anti-rabbit antibody (Sigma-Aldrich; diluted 1:10,000). Following the steps described above, the membrane was washed three times with PBST, treated with High-sig ECL Western Blotting Substrate, and developed on a Tanon 5200 (Tanon, Shanghai, China).

### Assessment of *in vitro* development of transgenic parasites by luciferase assay

HCT-8 cells were seeded on 48-well plates and grown to 70–80% confluence. INS6–3HA or *Δins6* oocysts (1 × 10^4^ oocysts per well) with similar luminescence values were used to infect the HCT-8 monolayer for 3, 12, 24, 36, and 48 h. At 2 hours post infection, cells were washed twice with PBS and replenished with fresh medium containing 2% fetal bovine serum to remove uninvaded parasites. At each time point, each well was washed with PBS and lysed with 100 µL lysis buffer, followed by incubation at 37°C for 10 min. Lysates were collected by centrifugation at 15,000 × g for 3 min and analyzed by luciferase assay as described above.

### Assessment of pathogenicity of INS6–3HA and *Δins6* strain in mice

To evaluate the effect of *INS6* depletion on parasite burden and pathogenicity, *Ifng*^−/−^ mice (3–5 weeks old) were divided into two groups: INS6–3HA and *Δins6*. Each mouse was orally gavaged with 1 × 10^3^ transgenic oocysts. After infection, mice were housed individually in micro-isolators with air filters to prevent cross-infection. Fecal pellets were collected every two days post-infection and stored at 4°C for luciferase assay and qPCR analysis. Body weight was recorded every two days, and survival was monitored until 40 dpi.

### Light and electron microscopy analyses

*Ifng*^−/−^ mice infected with INS6–3HA or *Δins6* strain were euthanized at 18 dpi, and ileal tissues were collected and fixed in 4% paraformaldehyde for 24 h and then transferred to 70% ethanol. Tissue sections were embedded in paraffin, sectioned, and stained with hematoxylin-eosin for light microscopy. For electron microscopy, some tissues were fixed in 2.5% buffered glutaraldehyde and processed for SEM using an EVO MA 15/LS 15 (Carl Zeiss AG, Oberkochen, Germany), while others were fixed in 4% paraformaldehyde and analyzed by IEM using a Talos L120C (ThermoFisher Scientific) as described [21]. For IEM, rabbit anti-HA (Cell Signaling Technology; diluted 1:20) and goat anti-rabbit IgG conjugated with 10 nm colloidal gold (Sigma-Aldrich; diluted 1:20) were used as primary and secondary antibodies, respectively.

### U-ExM analysis of sporozoites

U-ExM was performed on *Cryptosporidium* sporozoites as described for *Toxoplasma gondii* [25]. Briefly, excysted sporozoites were added to poly-D-lysine-coated coverslips and treated with a mixture of 1.4% (v/v) formaldehyde and 2% (v/v) acrylamide in PBS. The samples were embedded in a water-based gel, denatured at 95°C, and shrunk in PBS. The gels were probed with rat monoclonal anti-HA antibody (3F10; Roche; diluted 1:200) as primary antibody and goat anti-rat IgG (H&L) AF488 (Abcam; diluted 1:200) as secondary antibody. Parasite organelles were directly stained using NHS ester (Sigma-Aldrich; 10 μg/mL), and nuclei were stained with Hoechst (5 μg/mL). Images were acquired using a STELLARIS 5 (Leica Microsystems) and analyzed with ZEN 2 software (Zeiss).

### Statistical analysis

All data are presented as mean ± SD from at least three biological replicates. Statistical analysis was performed using GraphPad Prism 9.5.0. Specific statistical tests and *P* values are provided in the figure legends. No data were excluded from analysis, and *P* values < 0.05 were considered statistically significant.

## Results

### Endogenous tagging of *C. parvum* INS6

**Fig 1:**
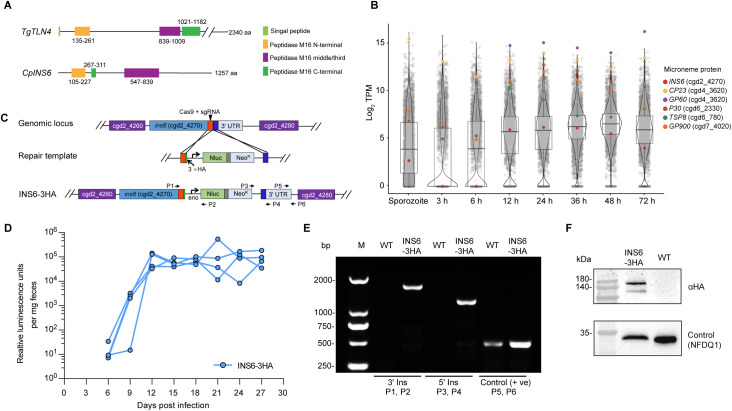
Endogenous epitope tagging of *INS6.* (A) Schematic representation of the structural domains of INS6 and its homologue gene, toxolysin 4 (TLN4). Both INS6 and TLN4 have three structural domains associated with classical insulinase peptidase, including the M16 N-terminus, the M16 middle/third terminus, and the M16 C-terminus, in addition to a signal peptide in TLN4. (B) Violin plots demonstrate the transcript expression of *C. parvum* genes in sporozoites and different development stages of parasites grown on HCT-8 cells, as indicated by TPM values from RNA-seq analysis of the transcriptome. Colored dots represent microneme genes, with *INS6* indicated in purple. (C) Schematic showing the tagging strategy of *INS6* locus. The location of the sgRNA, Cas9 break site, and the repair template for homologous recombination are shown. 3 × HA, triple hemagglutinin epitope tag; Nluc, nanoluciferase; Neo^R^, neomycin resistance marker; *Eno*, enolase promoter. (D) Fecal luminescence measurements of mice infected with INS6-3HA sporozoites. *Ifng*^−/−^ mice were infected with INS6-3HA electroporated sporozoites, relative luminescence was measured from feces after infection at indicated days. Each data point represents one mouse. In total, four mice were used in this experiment. (E) PCR analysis of INS6-3HA strain. Fecal genomic DNA from wild-type (WT) and INS6-3HA parasites were used as template, and primers for checking 5’ insertion (5’ Ins), 3’ insertion (3’ Ins), and the 3’ UTR sequence of INS6 as a positive control (+ve) are indicated in panel C. (F) Western blot analysis of INS6 expression in oocysts. INS6-3HA transgenic and WT oocysts were probed with anti-HA antibody (top panel) and a polyclonal antibody against the NFDQ1 as a loading control (bottom panel). NFDQ1 is a protein that contains the characteristic NFDQ amino acid repeat motifs [26].

Bioinformatic analysis indicated that *C. parvum* INS6 is a member of the secreted M16 metalloprotease family and shares three conserved domains with *Toxoplasma* toxolysin 4 (TLN4) (Fig 1A). However, sequence analysis of INS6 on CryptoDB indicates it is likely not a secretory protein, as it predicts an N-terminal transmembrane domain but no signal peptide. Transcriptomic analysis revealed minimal INS6 transcription during the early infection phase (3–6 h) and relatively low transcription during the middle to late phase (12–72 h) compared to other microneme proteins (Fig 1B). To investigate the subcellular localization of INS6, we generated a stable transgenic strain by adding a triple hemagglutinin (3 × HA) epitope tag to the C-terminus of the INS6, followed by the Nluc-Neo-Eno^R^ selection cassette using CRISPR/Cas9 genomic manipulation (Fig 1C). Excysted sporozoites were electroporated with INS6 tagging plasmid and Cas9 plasmid containing a gene-specific sgRNA targeting the *INS6* gene. Transfected sporozoites were used to infect four *Ifng*^−/−^ mice, with 16 g/L paromomycin selection administered in drinking water. Luminescence analysis showed that the transgenic parasites began shedding at 6 dpi, peaked at 12 dpi (Fig 1D). PCR analysis of fecal DNA confirmed the correct insertion of the 3 × HA tagging and selection cassette at the C-terminus of the the *INS6* gene (Fig 1E). Western blot analysis using an anti-HA antibody detected a ~140 kDa band in the INS6 lysate, which was absent in the wild-type sample, confirming INS6 expression (Fig 1F). Additionally, a smaller ~120 kDa band was observed, suggesting potential proteolytic processing of INS6.

### Low INS6 expression is accompanied by localization features that differ from those of typical microneme proteins

**Fig 2:**
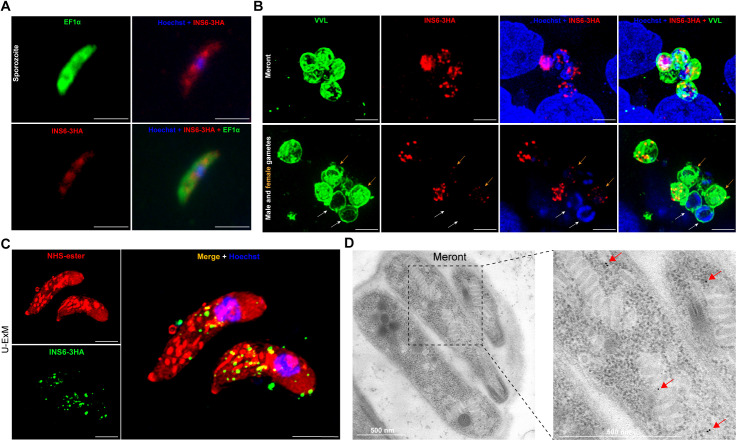
Localization and expression of *C. parvum* INS6. (A) Immunofluorescence assay of INS6-3HA expression in sporozoites. Parasites were fixed and stained with anti-HA (red) to label INS6 protein, anti-CpEF1α antibody (green) to label the entire parasite, and Hoechst to visualize parasite nuclei (blue). The INS6 protein was primarily localized to the mid-sporozoite region. Scale bars = 5 μm. (B) Immunofluorescence analysis of INS6-3HA expression in meronts, male gamonts and female gametes. Parasites were stained with anti-HA (red) to detect INS6 protein and VVL (green) to label the parasite. INS6 forms punctate aggregates in the meront, is not expressed in the male gamonts (indicated by the white arrow) and shows low expression in the female gametes (indicated by the orange arrow). Scale bars = 5 μm. (C) Ultrastructure expansion microscopy (U-ExM) of INS6-3HA in sporozoites. Sporozoites were fixed, expanded in gel, and stained with rat anti-HA (green), NHS-ester (red) and Hoechst (blue). The distribution pattern of INS6 exhibits partial spatial overlap with microneme proteins, but does not fully align with canonical microneme localization. Scale bars = 5 μm. (D) Immunoelectron microscopy of INS6-3HA. Mice were infected with INS6-3HA oocysts and the ileum tissues were fixed at 18 dpi and stained with rabbit anti-HA followed by 10-nm colloidal gold goat anti-rabbit IgG. Black dots indicate the distribution of gold particles, and red arrows indicate the distribution of INS6 protein.

To determine the stage-specific expression and localization of INS6, we performed immunofluorescence analysis on INS6–3HA strain. Immunofluorescence labeling revealed that INS6 is expressed at low levels during the sporozoite stage and is primarily localized to the mid-region of the sporozoite (Fig 2A). This is different from the apical localization of the microneme proteins gp40/gp900 and CpMIC2 [8]. During the meront stage, INS6 forms punctate aggregates within the meront (Fig 2B). Additionally, we investigated the expression of INS6 in male gamonts and female gametes. Using the nuclear morphology as described previously [27], we identified male gamonts and female gametes in infected samples cultured in HCT8 cells for 48 hours using immunofluorescence. We found that INS6 was not expressed in male gamonts and is only expressed at low levels in female gametes (Fig 2B). Using U-ExM, we observed INS6 associated with small uniform vesicles distributed in the middle half of sporozoites (Fig 2C). This distribution pattern differed from the canonical microneme localization pattern. This was further evidenced by concurrent signal detection within dense granule domains, demonstrating incompatibility with exclusive microneme localization. Furthermore, we performed immunoelectron microscopy (IEM) and detected gold particles distributed on micronemes in merozoites within meronts under the electron microscope (Fig 2D), due to the low expression levels, gold particles were observed in only a few micronemes.

### INS6 is not essential for *C. parvum* survival

**Fig 3:**
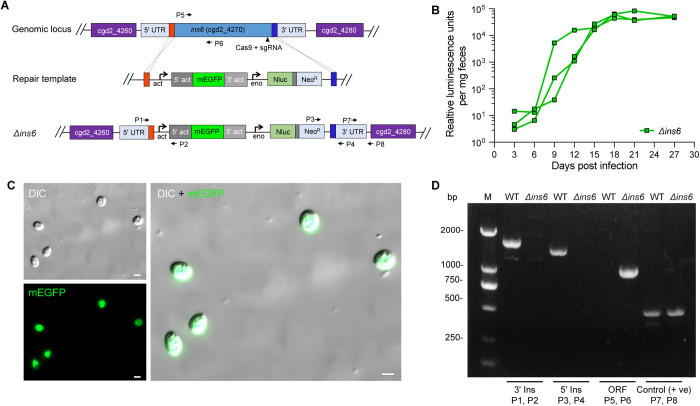
INS6 is not essential for parasite survival. (A) Schematic representation of the INS6 knockout strategy. The location of the sgRNA, the Cas9 cleavage site, and the repair template for homologous recombination are indicated. *pAct*, actin promoter. (B) Fecal luminescence measurements in mice infected with *Δ*ins6** sporozoites. *Ifng*^−/−^ mice infected with electroporated *Δ*ins6** sporozoites have their fecal samples analyzed for relative luminescence at the indicated time points post-infection. Each data point represents one mouse. In total, three mice were used in this experiment. (C) Fluorescence imaging of *Δ*ins6** oocysts. *Δ*ins6** oocysts expressing mEGFP were visualized using microscopy. Scale bars = 2 µm. (D) PCR analysis of the *Δ*ins6** strain. Genomic DNA extracted from fecal samples of WT and *Δ*ins6** parasites was used as a template. The primers used to verify the 5’ Ins, 3’ Ins, open reading frame (ORF) and 3’ UTR sequences of *INS6*, which were used as a positive control are indicated in panel A.

To investigate the essentiality of INS6 for *C. parvum* growth, we generated an INS6 knockout strain by replacing the *INS6* gene with an mEGFP expression cassette driven by the *C. parvum* actin promoter and a Nluc-Neo-Eno^R^ selection cassette using CRISPR/Cas9 (Fig 3A). Following transfection and selection in *Ifng*^−/−^ mice, analysis of fecal samples using luminescence confirmed the successful generation of the *Δins6* strain. For the *Δins6* strain, luminescence signals were first observed at 9 dpi, peaking at 18 dpi (Fig 3B). Both detection onset and peak attainment were significantly delayed relative to the INS6–3HA strain. Purified *Δins6* oocysts expressed GFP protein were observed by fluorescence microscopy (Fig 3C). PCR analysis confirmed the complete deletion of *INS6* and the correct insertions in the knockout cassette (Fig 3D). Comparative nanoluciferase assays showed similar growth between the INS6–3HA and *Δins6* strains, indicating that INS6 is not essential for parasite survival.

### INS6 regulates parasite growth *in vitro* and *in vivo*

**Fig 4:**
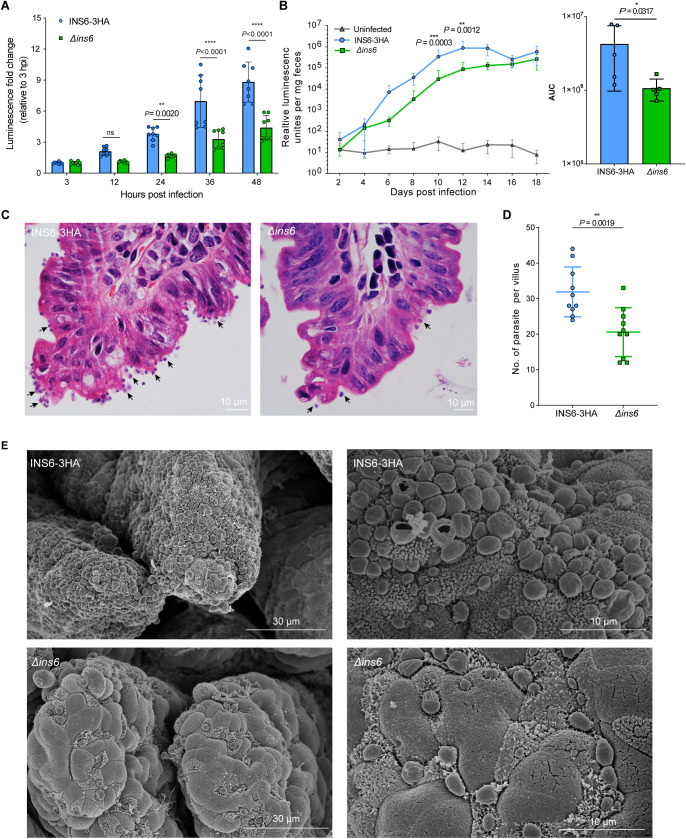
Genetic ablation of INS6 affects *C. parvum* proliferation. (A) Relative luminescence measurements of INS6-3HA and *Δ*ins6** parasites grown on HCT-8 cells. The cells were infected with either INS6-3HA or *Δ*ins6** parasites and the growth of the parasites was assessed by measuring luciferase activity at the indicated time points. Data are presented as the mean ± SD from eight replicates across two independent experiments. Differences between the INS6-3HA and *Δ*ins6** groups were analyzed using a two-way ANOVA followed by a Sidak’s multiple comparisons test. (B) Fecal luminescence measurements (left) and area under the curve analysis (right) in mice infected with INS6-3HA and *Δ*ins6** oocysts. *Ifng*^−/−^ mice were orally gavaged with either INS6-3HA or *Δ*ins6** oocysts, and parasite burden was quantified by luciferase activity at different time points. Data points represent mean ± SD from six or five individually housed infected mice (n = 6 for INS6-3HA and n = 5 for *Δ*ins6**). Differences between the INS6-3HA and *Δ*ins6** groups were analyzed using a two-way ANOVA followed by a Sidak’s multiple comparisons test. (C) Hematoxylin and eosin (H&E) staining of ileum sections from mice infected with INS6-3HA or *Δ*ins6** parasites. Black arrows indicate sites of *C. parvum* infection. (D) Quantification of parasite numbers per villus in the small intestine of infected mice. Each point on the graph represents the number of parasites on a single villus. Different shapes indicate individual data points. Select ten villi at random from each group and count the number of parasites on each one. Differences between the INS6-3HA and *Δ*ins6** groups were analyzed using two-tailed Mann-Whitney U test. (E) Scanning electron microscopy (SEM) images of the ileum from mice infected with INS6-3HA (top) or *Δ*ins6** (bottom) parasites.

To further assess the growth of the *Δins6* strain, we compared the luciferase activities of the *Δins6* and INS6–3HA strains in HCT-8 cells. During the early stages of asexual development (3–12 hpi), the absence of *INS6* is not affect the proliferation of *Cryptosporidium*, consistent with its low expression during the initial stages of infection (Fig 4A). However, after 24 hpi, when INS6 expression increased, the *Δins6* strain showed significantly reduced proliferation (Fig 4A), indicating a potential role for INS6 in asexual development.

*In vivo*, *Ifng*^−/−^ mice infected with *Δins6* oocysts showed significantly lower fecal luminescence at 10 dpi and 12 dpi compared to those infected with the INS6–3HA strain, with a nearly 10-fold decrease observed between 6 and 14 dpi (Fig 4B). However, this difference gradually diminished at later stages of the infection. This transient phenotype may be due to the high susceptibility of *Ifng*^⁻^^/^^⁻^ mice, as well as compensatory parasite expansion during iterative replication cycles, which progressively masks the initial growth defect caused by INS6 deletion. Histological examination of the ileum revealed fewer parasites on the villus surface in *Δins6*-infected mice than in INS6–3HA-infected mice (Fig 4C). Quantification confirmed a significantly higher parasite load in the INS6–3HA group (Fig 4D), a finding that is further supported by SEM (Fig 4E). These results suggest that INS6 depletion reduces parasite proliferation *in vivo*.

### INS6 depletion reduces *C. parvum* pathogenicity

**Fig 5:**
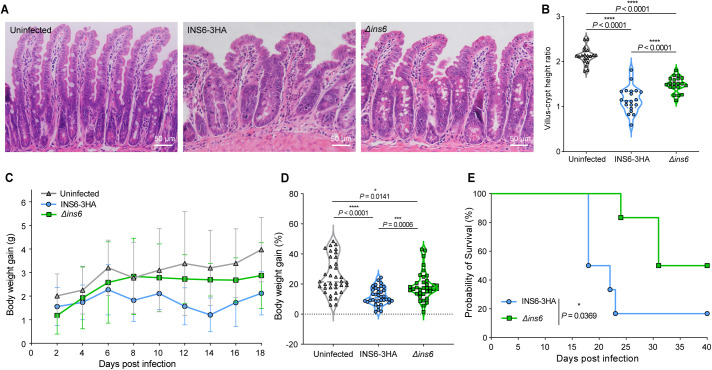
INS6 affects the pathogenicity of *C. parvum.* (A) Hematoxylin and eosin staining showing pathological damage to the ileum of *Ifng*^−/−^ mice infected with INS6-3HA or *Δ*ins6** strains. Uninfected mice were used as a control. (B) The ratio of villus height to crypt depth in mice infected with INS6-3HA, *Δ*ins6** strains or uninfected (two mice per group). (C) Body weight gain (g) in *Ifng*^−/−^ mice infected with INS6-3HA or *Δ*ins6** strains, with uninfected mice used as the control. Data were normalized to the day before infection. (D) Body weight gain (%). Data were collected from the day before infection until the end of the experiment. Five mice per group are shown in panels C and D. Differences among the INS6-3HA, *Δ*ins6** and uninfected groups were analyzed using two-way ANOVA followed by a Tukey’s multiple comparisons test in panels B and D. (E) Survival curves of *Ifng*^−/−^ mice infected with the INS6-3HA or *Δ*ins6** strains, or left uninfected as a control group. Data were obtained from five individually housed infected mice. Statistical significance was tested using the log-rank test.

We compared the pathological effects of the INS6–3HA and *Δins6* strains. H&E-stained ileal sections from INS6–3HA-infected mice showed significant villous atrophy, and shorter villi than those from *Δins6*-infected mice (Fig 5A). Quantification revealed a significantly lower villus/crypt ratio in INS6–3HA-infected mice than in *Δins6*-infected and uninfected controls (Fig 5B). Analysis of body weight showed that INS6–3HA-infected mice began to lose weight at 6 dpi, while *Δins6*-infected mice maintained higher body weights (Fig 5C). The *Δins6* group gained significantly more weight than the INS6–3HA group (Fig 5D). Depletion of INS6 delayed lethality in infected mice (Fig 5E), suggesting that INS6 contributes to *C. parvum* pathogenicity by promoting parasite growth *in vivo*.

## Discussion

INS6 is an insulinase-like protease belonging to the M16 family of metalloproteinases, characterized by an active domain containing the catalytic site. In this study, we showed that low levels of INS6 expression may be as a microneme protein involved in the pathogenic process of the parasite within its host. Unlike INS1, which is exclusively expressed in female gametes [21], INS6 expression is absent in male gamonts and present at low levels in female gametes. Its primary expression occurs in meronts, where it localizes on *C. parvum* micronemes and undergoes post-translational modification. Although INS6 is not expressed during the early stages of intracellular infection (0–6 hpi), *in vitro* proliferation experiments demonstrated that its absence impairs asexual stage proliferation and may reduce merozoite invasion efficiency. Furthermore, INS6 depletion led to reduced oocyst shedding and lower parasite loads on the intestinal epithelium *in vivo*. These findings suggest that INS6 plays a role in merozoite invasion during the asexual reproductive stage and modulates parasite pathogenicity.

INS6 may be transported by a non-canonical secretion pathway. Micronemes contain proteins such as perforins, adhesins and proteases, and their transport and secretion are critical for regulating invasion, egress, intracellular replication and virulence [28]. For example, the microneme protein CpTSP4 is secreted during *C. parvum* invasion and relies on kinesin-driven microtubule transport, indicating that microneme protein secretion may involve specific organelle-protein interactions [29]. In apicomplexan parasites, micronemes move through the conoid to the parasite apex, where they fuse with the plasma membrane to release their contents [30]. Sequence analysis of INS6 revealed an N-terminal transmembrane domain despite the absence of a signal peptide. This structural feature suggests a hypothetical model whereby INS6 may undergo non-canonical secretion, potentially utilizing the transmembrane domain as a signal anchor for membrane-associated trafficking [31]. Western blot analysis of 3 × HA-tag INS6 indicated potential post-translational proteolytic processing. Similar to *T. gondii* toxolysin-1, which is processed and transferred to the rhoptries during parasite-host interactions [11,32], INS6 may undergo analogous processing. Additionally, the *T. gondii* subtilisin-like protease (TgSUB1) is localized to micronemes, processed in the endoplasmic reticulum, and secreted in a calcium ion-dependent manner [33]. Future study will focus on elucidating the transport and secretion mechanisms.

The localization of INS6 differs from that of classic microneme proteins*. Cryptosporidium* possesses specialized secretory organelles, including micronemes, rhoptries, dense granules, and small granules [8]. Microneme proteins are primarily found in the anterior region of sporozoites and are associated with invasion [34,35]. Several microneme proteins, including CpGP900 (cgd7_4020), TRAPC1 (cgd1_3500), CpTSP4 (cgd8_150), CpMIC1 (cgd6_780), CpMIC2 (cgd7_1960), CpROM1 (cgd3_980), cgd1_3550, and cgd2_1590, have been experimentally confirmed to localize to the sporozoite anterior [8,29,36]. Among these, CpMIC2 was recently identified as part of the “microneme cluster” using HyperLOPIT technology, and this was confirmed by IFA, IEM and U-ExM [8]. HyperLOPIT analysis also identified INS6 as a microneme protein candidate and a homologue of *T. gondii* TLN4. Furthermore, single-cell RNA sequencing datasets (the GEO under accession number: GSE129267 and GSE232438) indicated that INS6 is co-expressed with other microneme proteins on the CryptoDB (https://CryptoDB.org/) [9]. Unlike CpTSP4, which forms filaments in the sporozoite [29], INS6 is localized to the mid-anterior region, which differs from the location of typical microsomal proteins. Small amounts of INS6 protein were also detected on the micronemes. However, due to its low expression level, the precise localization of INS6 cannot be fully determined. In subsequent studies, we will attempt to determine the subcellular localization of INS6 more precisely, either by overexpressing it or by co-localizating it with a microneme marker.

INS6 may influence *C. parvum* merozoite invasion efficiency and participate in proliferation. Microneme proteins are essential for producing invasive merozoites and sporozoites, and their transcriptional patterns correspond to the biogenesis of organelles involved in the invasion process [8]. Microneme proteins regulate the invasion of host cells by apicomplexan parasites in multiple ways. For example, *P. falciparum* CLAMP proteins regulate the secretion of adhesion factors in malaria sporozoites [37], while *T. gondii* MIC2 proteins mediate parasite gliding motility and their initial contact with host cells [38]. The *T. gondii* homologue of INS6, TLN4, is involved in parasite invasion and egress from host cells [10,11]. INS6 transcription occurred at the end of the asexual replication cycle (24 hpi) and was essentially not expressed during the sexual stage. This is consistent with previous single-cell sequencing results [9]. *In vitro* growth comparison between *Δins6* and INS6–3HA strains showed significantly reduced proliferation of the *Δins6* strain at 24 hpi, suggesting a role in merozoite invasion or egress efficiency. In the mouse infection model, INS6–3HA parasites exhibited a 10-fold increase in oocyst shedding and higher villus surface parasite loads compared to *Δins6* parasites. These results indicate that INS6 plays a key role in parasite growth, particularly during asexual reproduction. However, this study was unable to determine the precise mode of action of INS6 in specific developmental stages such as meronts, gamonts and oocyst production. Given the urgent need for effective therapeutics against *C. parvum*, further investigation of INS6 may provide a mechanistic foundation for developing novel drug targets or transmission-blocking interventions.

Depletion of INS6 attenuates the pathogenicity of *C. parvum*. The pathogenicity of *C. parvum* varies based on the source and subtype of the isolate. In this study, the wild-type *C. parvum* isolate (IIdA20G1-HLJ) originated from a diarrhea outbreak on a dairy farm that resulted in the death of approximately 60 calves [15]. In the mouse infection model, *Ifng*^−/−^ mice are highly susceptible to *C. parvum* infection and develop severe gastrointestinal disease, characterized by extensive infection of lower intestinal epithelial cells and severe mucosal damage, and death within 2–4 weeks [16,39]. The IIa subtype is the most prevalent *C. parvum* strain in developed countries, and the AUCP-1 strain is highly pathogenic [21,40]. Deletion of CDPK5 in the AUCP-1 strain reduces male gamonts production and oocyst shedding, attenuating virulence in *Ifng*^−/−^ mice [41]. Similarly, depletion of INS6 in IIdA20G1-HLJ attenuated weight loss, reduced intestinal damage, and delayed lethality in infected *Ifng*^−/−^ mice. These findings suggest that INS6 modulates host clinical phenotypes during *C. parvum* infection, potentially altering pathogenesis.

In conclusion, our findings demonstrate that INS6 plays a role in the asexual proliferation stage of *C. parvum* by influencing oocyst formation and reducing parasite loads on intestinal epithelial cells, ultimately attenuating pathogenicity. These insights advance our understanding of *C. parvum* biology and may aid in identifying potential drug targets and understanding pathogenesis mechanisms.

## Supporting information

S1 Table PCR primers used in this study. (PDF)

S2 Table Source data. (XLSX)
