# Supplementary material for: Cryptosporidium parvum protease INS6 plays an important role in parasite proliferation and pathogenicity
Source: PLoS Negl Trop Dis. 2025 Sep 12;19(9):e0013532. doi: 10.1371/journal.pntd.0013532 (PMC12443312; doi:10.1371/journal.pntd.0013532)
Supplement: S1 Table — (PDF) [file pntd.0013532.s001.pdf]

Table S1 - Primers used in this study.

Primer Name	Sequence (5' - 3')	Usage
tracr RNA-R1<br>U6 F	GTTTTAGAGCTAGAAATAGCAAG<br>CCCAACACTTAACCTTTCAGT	Primer set used to amplify Cas9 plasmid backbone (9963 bp) for building pACT1:Cas9-GFP, U6:sgINS6
INS6-gRNA-linker	CTGAAAGGTTAAGTGTTGGGAAAAT GCAAGCTTTGCAAGGTTTTAGAGCTA GAAATAGC	Primer set used to build pACT1:Cas9-GFP, U6:sgINS6 by Gibson assembly
INS6-C-Tagging-F<br>INS6-C-Tagging-R	AGCTGAGCTCCGCAGTAGTAGCTCACT<br>CCTTGCTAGCAAGCTTGCTATGATCGC	Primer set used to amplify INS6 C-terminus homology arm (994 bp) for building pINS6-3HA-Nluc-P2A-neo by Gibson assembly
INS6-3' UTR-Tagging-F<br>INS6-3' UTR-Tagging-R	CTTCTGATTACGTAATAATTCATTTAG GTATTGC<br>GAGGATCTCCCCTTTTTCGTAAAAGGA GTC	Primer set used to amplify INS6 3' UTR homology arm (952 bp) for building pINS6-3HA-Nluc-P2A-neo by Gibson assembly
INS6 Nluc-Tagging-F<br>INS6 Nluc-Tagging-R	TAGCAAGCTTGCTAGCAAGGGCTCGG GC<br>AATTATTACGTAATCAGAAGAATTCGT CAAGAAGACGATAGAAGG	Primer set used to amplify 3HA-Nluc-P2A-neo casstte (2104 bp) for building pINS6-3HA-Nluc-P2A-neo by Gibson assembly
INS6-pUC19 -Tagging-F<br>INS6-pUC19-Tagging-R	ACGAAAAAGGGGAGATCCTCTAGAGT CG<br>TACTACTGCGGAGCTCAGCTCGAATT CAC	Primer set used to amplify plasmid backbone (2704 bp) for building pINS6-3HA-Nluc-P2A-neo by Gibson assembly
INS6 5' UTR-KO-F<br>INS6 5' UTR-KO-R	AGCTGAGCTCTGCTATCGCCCACGTC GC<br>ATAAGAAATTCTCAAGATTTCTTTCAAT GCTTTTCAATTTCCC	Primer set used to amplify INS6 5' UTR homology arm (993 bp) for building INS6-GFP-Nluc-P2A-neo-INS6 by Gibson assembly
INS6-3' UTR-KO-F<br>INS6-3' UTR-KO-R	AGGAGAAGAGCGTAATAATTCATTTAG GTATTGC<br>AGCTGGTACCCCTTAAAAGTTTCGTAA TCG	Primer set used to amplify INS6 3' UTR homology arm (990 bp) for building INS6-GFP-Nluc-P2A-neo-INS6 by Gibson assembly
INS6-Nluc-KO-F<br>INS6 Nluc-KO-R	AAATCTTGAGAATTTCTTATCAAACTC AAGTTAAAAAG<br>AATTATTACGCTCTTCTCCTTTTCCATA AAAG	Primer set used to amplify GFP-Nluc-P2A-neo casstte (4540 bp) for building INS6-GFP-Nluc-P2A-neo-INS6 by Gibson assembly
INS6-pUC19-KO-F<br>INS6-pUC19-KO-R	ACTTTTAAGGGGTACCAGCTCGGTAC CC<br>GGCGATAGCAGAGCTCAGCTCGAATT CAC	Primer set used to amplify plasmid backbone (2722 bp) for building INS6-GFP-Nluc-P2A-neo-INS6 by Gibson assembly
INS6-PAM-F<br>INS6-PAM-R	CGGAAAAGATTCAAAGGAGGTG<br>TTCGGAAATATTACATTGAATATCCG	Primer set used to mutate PAM sequence of INS6 gRNA in pINS6-3HA-Nluc-P2A-neo
INS6-gRNA-linker1	CAATGTAATATTTCCGAAGAAAATGCA AGCTTTGCAAGCGGAAAAGATTCAAA GGAG	Primer set used to build pINS6-3HA-Nluc-P2A-neo with mutate PAM sequence of INS6 gRNA by Gibson assembly

INS6-Tag-N-Detection-F<br>INS6-Tag-N-Detection-R	GCTCTTATTGTAGGCAACATTGG<br>ATCATAGGGATAGCCAGCGTAG	Primer set “5' Ins” used to detect INS6-3HA insection site, WT = null; 3HA tagging =1242 bp
INS6-Tag-C-Detection-F<br>INS6-Tag-C-Detection-R	ATGGCTGATGCTATGCGTC<br>CCGTTTCTAAGTGGTTGTTCG	Primer set “3' Ins” used to detect INS6-3HA insection site, WT = null; 3HA tagging =1679 bp
INS6-KO-N-Detection-R<br>INS6-KO-N-Detection-R	GACAAACAAGGCTTTCCAATG<br>GCCCTAACGCTTTCTATTATTACTG	Primer set “5' Ins” used to detect *Δins6* spanning the 5' CRISPR targeting site, WT = null; *Δ ins6* = 1384 bp
INS6-KO-C-Detection-F<br>INS6-KO-C-Detection-R	ACTGTGGTCGTCTTGGTGTC<br>CCGTTTCTAAGTGGTTGTTCG	Primer set “3' Ins” used to detect Δ ins6 spanning the 3' CRISPR targeting site, WT = null; Δins6 = 1608 bp
WT-INS6-F<br>WT-INS6-R	ACTTTGAGTGGGAGGAAG<br>AGCGAGTCAGATACAAGC	Primer set used to detect the wild-type locus, WT = 492 bp; 3HA tagging =492; *Δins6* = 492 bp
18S-LC2-F2<br>18S-LC2-R2	AAGTATAAACCCCTTTACAAGTA<br>TATTATTCCATGCTGGAGTATTC	Primer set used to quantify oocysts oocysts number by qPCR
